# Antagonizing Activin A/p15^INK4b^ Signaling as Therapeutic Strategy for Liver Disease

**DOI:** 10.3390/cells13070649

**Published:** 2024-04-08

**Authors:** Sowmya Mekala, Ravi Rai, Samantha Loretta Reed, Bill Bowen, George K. Michalopoulos, Joseph Locker, Reben Raeman, Michael Oertel

**Affiliations:** 1 Department of Pathology, Division of Experimental Pathology, University of Pittsburgh, 200 Lothrop Street—BST S-404, Pittsburgh, PA 15261, USAraviprakash.rai@pitt.edu (R.R.); michalopoulosgk@upmc.edu (G.K.M.); reben.raeman@pitt.edu (R.R.); 2 Pittsburgh Liver Research Center (PLRC), University of Pittsburgh, Pittsburgh, PA 15261, USA; 3 McGowan Institute for Regenerative Medicine, University of Pittsburgh, Pittsburgh, PA 15219, USA

**Keywords:** liver regeneration, cell cycle arrest, senescence, activin antagonist, liver fibrosis

## Abstract

Background/Aim: Activin A is involved in the pathogenesis of human liver diseases, but its therapeutic targeting is not fully explored. Here, we tested the effect of novel, highly specific small-molecule-based activin A antagonists (NUCC-474/555) in improving liver regeneration following partial hepatectomy and halting fibrosis progression in models of chronic liver diseases (CLDs). Methods: Cell toxicity of antagonists was determined in rat hepatocytes and Huh-7 cells using the 3-(4,5-dimethylthiazol-2-yl)-2,5-diphenyl-2H-tetrazolium bromide assay. Hepatocytes and hepatic stellate cells (HSCs) were treated with activin A and NUCC-555 and analyzed by reverse transcription–polymerase chain reaction and immunohistochemistry. Partial hepatectomized Fisher (F)344 rats were treated with NUCC-555, and bromodeoxyuridine (BrdU) incorporation was determined at 18/24/36/120/240 h. NUCC-555 was administered into thioacetamide- or carbon tetrachloride-treated F344 rats or C57BL/6 mice, and the fibrosis progression was studied. Results: NUCC-474 showed higher cytotoxicity in cultured hepatic cells; therefore, NUCC-555 was used in subsequent studies. Activin A-stimulated overexpression of cell cycle-/senescence-related genes (e.g., *p15^INK4b^*, *DEC1*, *Glb1*) was near-completely reversed by NUCC-555 in hepatocytes. Activin A-mediated HSC activation was blocked by NUCC-555. In partial hepatectomized rats, antagonizing activin A signaling resulted in a 1.9-fold and 2.3-fold increase in BrdU^+^ cells at 18 and 24 h, respectively. Administration of NUCC-555 in rats and mice with progressing fibrosis significantly reduced collagen accumulation (7.9-fold), HSC activation indicated by reduced alpha smooth muscle actin^+^ and vimentin^+^ cells, and serum aminotransferase activity. Conclusions: Our studies demonstrate that activin A antagonist NUCC-555 promotes liver regeneration and halts fibrosis progression in CLD models, suggesting that blocking activin A signaling may represent a new approach to treating people with CLD.

## 1. Introduction

The liver is an exceedingly complex organ with remarkable regenerative potential, regulating many essential physiological processes that require maintenance of a constant healthy liver mass. This organ harbors parenchymal (hepatocytes and cholangiocytes) and non-parenchymal cells (hepatic stellate cells (HSCs), endothelial cells, and Kupffer cells), essential for tissue growth and function [1,2]. Hepatocytes—normally in a non-proliferative (quiescent) state characterized by temporary cell cycle arrest [3]—are the main cells with regenerative potential, which is essential to re-enter the cell cycle and fully replace injured tissue mass (hepatostat). However, hepatotoxins, chronic injury, and age-related changes disrupt the hepatostat, resulting in impaired liver regeneration and hepatic function [4,5].

Cytokines and growth factors play a critical role during hepatocyte proliferation by regulating liver regeneration [6]. One of them is activin A, a multifunctional glycoprotein and member of the transforming growth factor (TGF)-β-superfamily [7]. In normal adult liver, activin A—primarily expressed in hepatocytes—acts as a growth-inhibitory cytokine and an inducer of senescence, as well as a regulator of metabolic function [8,9]. As a negative regulator of cell growth, activin A can block proliferation by inhibiting DNA synthesis or inducing apoptosis in hepatocytes [10,11,12]. The majority of biological effects of activin A in hepatocytes are Smad dependent and mediated [9]. Activin A binds the type II receptors Act-IIA and Act-IIB [13]. Activated type II receptors further phosphorylate and recruit the type I receptor ALK-4. Subsequently, activated type I receptors directly phosphorylate receptor-regulated Smads (Smad 2/3) in the cytoplasm for downstream signaling [14]. These phosphorylated R-Smads form heterocomplexes with the common Smad 4 before they translocate to the nucleus [15]. A major target gene of activin A signaling in hepatocytes is the cyclin-dependent kinase inhibitor p15^INK4b^, which mediates growth inhibition and induction of senescence [9,16].

Increased activin A levels in hepatocytes were observed in patients with alcoholic cirrhosis, acute liver failure, nonalcoholic fatty liver disease (NAFLD), chronic viral hepatitis, and hepatocellular carcinoma (HCC), implying a critical role of activin A in the pathogenesis of human liver disorders [7,17,18,19,20,21,22,23]. However, therapeutic targeting of activin A has not yet been fully explored. There are two natural antagonists of activin A. With a short half-life time, inhibin serves as a competitive antagonist of activin by binding type II activin receptors; however, betaglycan is required to promote inhibin antagonism [24,25]. Follistatin has a high binding affinity to activin A [26], but also to other molecules, e.g., bone morphogenic proteins (BMPs)-2/4/7 [27,28]. SB-431542 and SB-505124 are potent ALK-4 inhibitors, but they also bind the type I receptors ALK-5 and ALK-7 [29,30]. Because of the low specificity of these inhibitors, their clinical potential is limited, and, therefore, novel therapeutic strategies specifically targeting activin A signaling are urgently needed in patients with liver dysfunction associated with chronic liver diseases (CLDs).

In the present study, we have investigated the effect of a novel highly specific small-molecule-based activin A antagonist. Previous studies [24] identified an activin A-binding pocket between both βA-subunits, which disrupts the activin A/receptor interaction. Using an in silico screening of an 18 million compound ZINC database, the authors identified two potential lead molecules (NUCC-474, NUCC-555). We investigated the cell toxicity of both compounds in hepatic cells and selected NUCC-555 for subsequent studies. NUCC-555 completely blocks activin A-mediated overexpression of cell cycle- and senescence-related target genes in hepatocytes. Our experiments demonstrated that blocking the activin A/p15^INK4b^ signaling axis increases hepatocyte proliferation in the liver after partial hepatectomy (PH), which led us to study the effect of NUCC-555 on the progression of fibrosis in two rodent models of CLD.

## 2. Materials and Methods

### 2.1. Animals

Male dipeptidyl-peptidase IV-positive (DPPIV^+^) Fisher (F)344 rats were purchased from Charles River (Wilmington, MA, USA), and DPPIV^–^ F344 rats were originally obtained from the Rat Resource and Research Center, University of Missouri-Columbia, and maintained at the University of Pittsburgh. In the present study, F344 rats were used for hepatocyte isolation, used in the in vitro experiments, and in vivo studies. C57BL/6 mice were purchased from Jackson Laboratories (Bar Harbor, ME, USA), bred and maintained at the University of Pittsburgh. All animal studies were performed according to the animal protocols approved by the Institutional Animal Care and Use Committees of the University of Pittsburgh in accordance with National Institutes of Health (NIH) guidelines.

### 2.2. Activin A Antagonists

The small-molecule compounds NUCC-475 and NUCC-555 were purchased from ChemBridge Corp. (San Diego, CA, USA) and dissolved in dimethyl sulfoxide (DMSO).

### 2.3. Isolation and Purification of Rat Hepatocytes

Detailed information can be found in the Supplemental Material and Methods.

### 2.4. Rat Hepatic Stellate Cells (HSCs)

Rat-activated HSCs were purchased from ScienCell Research Laboratories (Carlsbad, CA, USA). Cells were isolated from postnatal day 2 rat livers and cryopreserved after purification and delivered frozen. After thawing, cells were cultured and expanded in a stellate cell medium (SCM; ScienCell). Passages 4–6 were used for experiments (see Section 2.5.2).

### 2.5. Cell Culture Experiments

#### 2.5.1. Rat Hepatocytes

Freshly isolated and purified 1 × 10^5^ rat hepatocytes were plated on collagen-coated 6-well plates (9.07 cm^2^/well) and incubated in Dulbecco’s modified eagle medium (DMEM) with 10% fetal bovine serum (FBS) at 37 °C overnight (O/N). The next day, the culture medium was switched to a hormonally defined growth medium (HGM) [31], followed by incubation for 24 h. At day 3, hepatocytes were treated without or with various concentrations of NUCC-474 or NUCC-555 (0.1, 0.5, 2.5, 12.5, 62.5 µg/mL) for 48 h, followed by incubation with 3-(4,5-dimethylthiazol-2-yl)-2,5-diphenyltetrazolium bromide) (MTT; MTT assay, see Section 2.6). In additional experiments, starting at day 3, hepatocytes were incubated without or with 50 ng/mL recombinant activin A (StemCell Technologies, Vancouver, Canada) and various concentrations of NUCC-555 (0.5 and 5.0 µg/mL) for 48 h, followed by RNA extraction (qRT-PCR see Section 2.7). Cytospins were prepared at the rate of 5000 cells/slide, followed by immunohistochemistry (IHC) for alpha smooth muscle actin (α-SMA; see Section 2.11.3.).

#### 2.5.2. Rat HSCs

Rat-activated HSCs (1 × 10^5^ cells/9.07 cm^2^/well) were plated on 6-well plates and incubated in SCM at 37 °C for 5 days. To inactivate these cells, activated HSCs were plated on 6-well plates and cultured in SCM O/N. The next day, the culture medium was switched to Clevers’ medium (CM) [32], followed by incubation for 4 days. Clever’s medium was developed by the Hans Clever’s group for culturing mouse hepatocytes and generating organoids, as described in [32]. Dr. Michalopoulos’s group used this medium in their unpublished in vitro studies and discovered that activated HSCs can be deactivated in CM. In additional experiments, activated HSCs were plated in SCM O/N, followed by incubation in CM for 4 days. Inactivated HSCs were treated with 50 ng/mL activin A without or with 5 µg/mL NUCC-555 for 4 days, followed by RNA extraction. Cytospins were prepared and additional analyses were performed (immunocytochemistry for α-SMA, see Section 2.11.3).

#### 2.5.3. Human Huh-7 Cells

Human Huh-7 cells (kindly provided by Dr. Locker) were plated in 12-well plates (1 × 10^5^ cells/3.65 cm^2^/well) and cultured in DMEM containing 10% fetal FBS. At day 2, cells were treated without or with various concentrations of NUCC-474 or NUCC-555 (0.1, 0.5, 2.5, 12.5, 62.5 µg/mL) for 48 h, followed by incubation with MTT (MTT assay, see Section 2.6).

### 2.6. MTT Assay

The MTT assay determines the metabolic activity of viable, as well as proliferating cells. Rat hepatocytes and Huh-7 cells were incubated in their respective medium with various concentrations of NUCC-474 or NUCC-555 for 48 h (see Section 2.5.1 and Section 2.5.3). Cells without activin A antagonist treatment served as controls. Thereafter, the cell culture medium was replaced with a medium containing 0.8 mg/mL MTT, and cells were incubated at 37 °C for 4 h. In viable cells, the MTT was reduced to formazan. In the next step, formazan crystals were dissolved in a solubilization solution (sodium dodecyl sulfate-N, N-dimethylformamide [SDS-DMF]), followed by measurement of the absorbance at 570 nm, using the NanoDrop 2000c spectrophotometer (Thermo Electron North America LLC, West Palm Beach, FL, USA). In our studies, the MTT assay was used to determine the cytotoxicity of the compounds. The percentage of cytotoxicity was normalized to cultured cells without activin A antagonist treatment.

### 2.7. Quantitative Reverse Transcription-Polymerase Chain Reaction (qRT-PCR)

Total rat RNA was extracted from cultured hepatocytes, HSCs, or snap-frozen liver samples using trizol reagent (Life Technologies, Carlsbad, CA, USA). In cell culture experiments, 1 × 10^5^ cells/well were cultured in triplicates, cell extracts were pooled, and total RNA was isolated and used for PCR analyses. For RNA analysis of liver tissues, 3 small frozen liver pieces (1–2 mm lengths), derived from 3 different lobes per rat, were pooled and used for RNA isolation. In addition, liver samples were dissolved in trizol and treated with DNAse I (New England Biolabs, Ipswich, MA, USA), followed by cleanup using the RNeasy Plus Mini/Micro Kit (Qiagen, Germantown, MD, USA). Using the cDNA synthesis kit from Thermo Fisher (Waltham, MA, USA), extracted RNA was reverse transcribed. qRT-PCR analyses were performed in at least 3 independent experiments, each with duplicate assays, using the StepOnePlus Real Time PCR System (Applied Biosystems, Waltham, MA, USA). Samples were analyzed using Power SYBR Green Master Mix (Applied Biosystems). mRNA abundance was determined by normalization of the data to the expression levels of glyceraldehyde 3-phosphate dehydrogenase (Gapdh) mRNA. A complete list of rat primers is shown in Table S1.

Total mouse RNA was isolated from liver tissues, followed by cDNA synthesis and qRT-PCR analyses, based on previously described methods [33]. Data were normalized against 18S rRNA and fold change in gene expression compared to controls was reported. For primers, see Table S1.

### 2.8. PH, NUCC-555 Treatment, and Bromodeoxyuridine (BrdU) Administration in Rats

Two-thirds PH was performed as described [34]. NUCC-555 was administered intraperitoneally (50 mg/kg body weight [b.w.]; dosage by Zhu et al. [24]) one day prior to and subsequently after PH, and on days 2, 4, 6, and 8, thereafter. Two hours prior to sacrifice, BrdU (100 mg/kg b.w.) was injected intraperitoneally into rats. Animals were sacrificed at various time points and liver tissues were harvested for further analyses.

### 2.9. Thioacetamide (TAA)-Induced Liver Fibrosis and NUCC-555 Administration in Rats

Two hundred mg/kg b.w. TAA was injected intraperitoneally into F344 rats (1.5 to 2 months of age) twice weekly for 10 weeks. After 2 weeks, NUCC-555 (50 mg/kg b.w.; dosage by Zhu et al. [24]) treatment was started and continued thereafter, twice a week. The control group received an equal volume of vehicle solution.

### 2.10. Carbon Tetrachloride (CCl_4_)-Induced Liver Fibrosis and NUCC-555 Administration in Mice

Male C57BL/6 mice received CCl_4_ (2 mL/kg b.w., 1:1 [*v*/*v*] mixture with olive oil) or vehicle gavage twice weekly for 6 weeks. At two weeks after starting CCl_4_ administration, one cohort of mice receiving CCl_4_ was treated with NUCC-555 (30 or 60 mg/kg b.w. NUCC-555, two times per week; doses Zhu et al. [24]), and the second cohort an equal volume of vehicle control by intraperitoneal injection for four weeks.

### 2.11. IHC/Histochemistry/Immunocytochemistry

#### 2.11.1. Nuclear staining for BrdU

Detailed information can be found in the Supplemental Material and Methods.

#### 2.11.2. Collagen Staining

Detailed information can be found in the Supplemental Material and Methods.

#### 2.11.3. Immunohistochemical/Immunocytochemical Detection of α-SMA and Vimentin

Detailed information can be found in the Supplemental Material and Methods.

### 2.12. Serological Analyses

Rat blood serum samples were analyzed for aspartate aminotransferase (AST), alanine aminotransferase (ALT), and alkaline phosphatase (ALP) by IDEXX BioAnalytics (Columbia, MO, USA). AST and ALT activity kits (TECO Diagnostics, Anaheim, CA, USA) were used to determine the serum aminotransferase activity in mouse serum.

### 2.13. Microscopy and Imaging

Microscopic images were acquired using an AxioCam HRc camera and further processed with ZEN pro 2012 imaging software, installed on an AxioObserver Z1 microscope (Carl Zeiss, Oberkochen, Germany). BrdU-positive cells and collagen-, α-SMA-, and vimentin-positive tissue areas were calculated using ImageJ software (NIH).

### 2.14. Statistical Data Analysis

The two-tailed Student’s *t*-test was used to assess the mean differences between two independent groups. For comparisons among multiple groups, one-way ANOVA followed by post hoc analysis was applied to determine statistical differences. A *p*-value of less than 0.05 was deemed to indicate statistical significance. The data presented are indicative of two to three separate experiments. All statistical evaluations were conducted using the GraphPad Prism version 8.0 software by GraphPad Software Inc., San Diego, CA, USA.

## 3. Results

### 3.1. Cytotoxicity of NUCC-474/555 in Human Huh-7 Cells and Rat Hepatocytes

**Figure 1:**
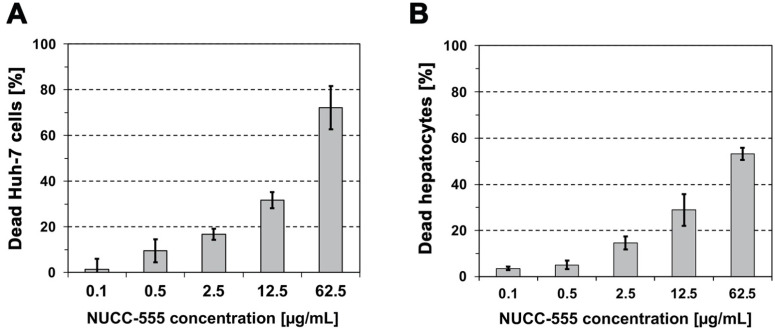
Cell toxicity of the activin A antagonist NUCC-555. Cells were plated on culture plates (1 × 10^5^ cells/well), followed by incubation with NUCC-555 for 48 h. Toxicity was determined by standard MTT assay. Cytotoxic effect of NUCC-555 at various concentrations in human Huh-7 cells (**A**) and primary rat hepatocytes (**B**). Each time point represents the mean ± SEM of three independent experiments, which were performed in triplicates. The percentage of cytotoxicity is normalized to cultured cells without activin A antagonist treatment.

Zhu et al. identified two novel highly specific small-molecule-based activin A antagonists and demonstrated that NUCC-474 and NUCC-555 effectively antagonized activin A in ex vivo ovary cultures and in ovariectomized mice [24]. To evaluate the antagonistic effects of these compounds in hepatic cells in vitro and in vivo, we first tested their cell toxicity in cultured Huh-7 cells and primary hepatocytes, using the MTT assay. Cells were treated with activin A antagonists at various concentrations for 48 h. Both cell types exhibited a similar dose-dependent increase in cell death after incubation with NUCC-474 (Supplemental Figure S1A,B) or NUCC-555 (Figure 1A,B). We observed lower cytotoxicity levels in Huh-7 cells and rat hepatocytes treated with NUCC-555 relative to NUCC-474. NUCC-555 killed less than 20% of both cell types within 48 h of incubation at a concentration of 2.5 μg/mL (Figure 1A,B); however, NUCC-474 killed 19.3 ± 8.9% of Huh-7 cells and 36.1 ± 4.5% of hepatocytes (Supplemental Figure S1A,B). The cytotoxicity levels in hepatocytes treated with 62.5 μg/mL NUCC-474 and NUCC-555 were 87.3 ± 1.7% and 53.2 ± 2.6%, respectively (Supplemental Figure S1B and Figure 1B). Because NUCC-474 treatment showed higher cytotoxic effects in hepatic cells in vitro, we used NUCC-555 in subsequent studies.

### 3.2. Blockage of Activin A-Induced Overexpression of Selected Genes by NUCC-555

In our previous studies, we reported many overexpressed genes after activin A treatment in cultured rat hepatocytes [9]. Most of those genes were related to cell cycle arrest/senescence but not to apoptosis. Therefore, to test the potential of NUCC-555 in blocking activin A effects in rat hepatocytes, we cultured hepatocytes with activin A with or without NUCC-555 and analyzed mRNA extracts for selected gene expression. The genes of interest were *p15^INK4b^*, deleted in esophageal cancer 1 *(DEC1)*, and galactosidase b 1 *(Glb1)* (key target genes of activin A signaling controlling proliferation/senescence in hepatocytes [9,16]. Because we also discovered several novel genes with different functions that are highly induced after activin A treatment in hepatocytes, we also tested the mRNA levels of ADAM metallopeptidase domain 12 *(Adam12)*, prostate transmembrane protein, androgen-induced 1 *(Pmepa1)*, Wnt family member 7a *(Wnt7a)*, LIM and cysteine-rich domain 1 *(Lmcd1)*, shroom family member 4 *(Shroom4)*, and transient receptor potential cation channel, subfamily M, member 4 *(Trpm4)* [9]. Hepatocytes were cultured in HGM to stimulate cell proliferation and subsequently treated with recombinant human activin A (50 ng/mL) without or with NUCC-555 (0.5 and 5.0 µg/mL). These antagonist doses were used based on our in vitro studies (see Figure 1) showing a low cytotoxicity within this treatment range.

**Figure 2:**
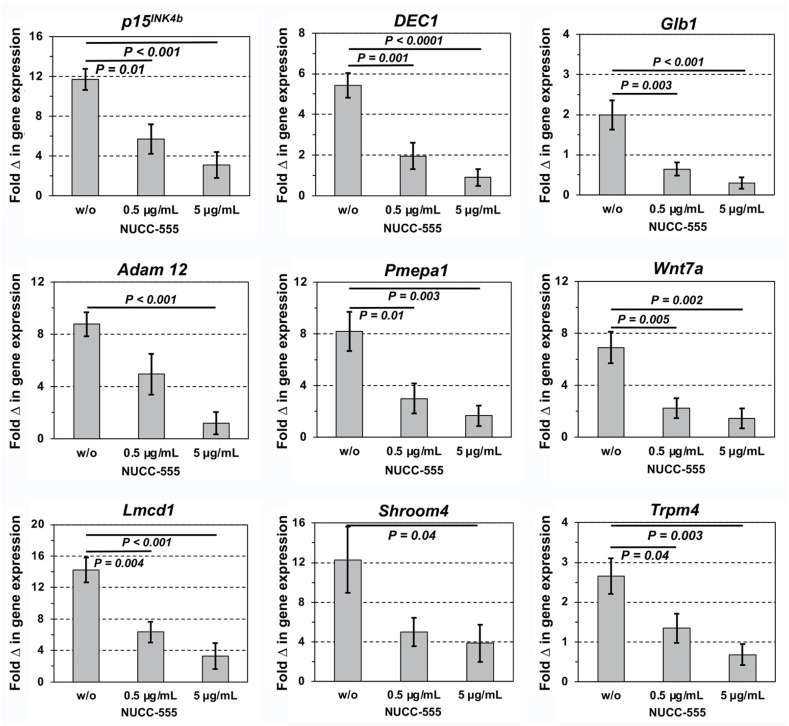
Inhibition of activin A-induced effects of mRNA expression in rat hepatocytes. Cells (1 × 10^5^ cells/well in triplicates) were treated with 50 ng/mL of recombinant activin A and exposed to various concentrations of NUCC-555 for 48 h. RNA extracts were analyzed by qRT-PCR and expression levels of selected genes were determined. Mean ± SEM values of three independent experiments (including two replicate PCR analyses, each) are expressed as fold changes with respect to cultured hepatocytes without activin A and antagonist, set at a value of 1.

A 11.7-fold gene expression increase of the activin A key target gene *p15^INK4b^* was reduced to 3.1-fold by NUCC-555 (Figure 2, upper-left panel), indicating that antagonizing activin A signaling effectively reverses impaired hepatocyte proliferation. Moreover, *Dec1* and *Glb1* were entirely reduced by 5 μg/mL of the activin A antagonist (Figure 2, upper-middle and right panels), demonstrating that NUCC-555 is capable of blocking activin A-mediated induction of senescence in hepatocytes. *Adam12*, *Pmepa1*, *Wnt7a*, *Lmcd1*, *Shroom4*, and *Trpm4* mRNAs were increased 2.7 to 14.2-fold with activin A, and effects were significantly reversed by blocking activin A using NUCC-555 (Figure 2, middle and lower panels). Collectively, our data provide strong evidence that activin A-stimulated gene overexpression can be effectively blocked in hepatocytes by the novel activin A antagonist NUCC-555.

### 3.3. Role of Activin A Signaling in Rat HSCs

Our data in Figure 2 showed that small-molecule-based blockage of activin A signaling inhibited activin A-induced gene overexpression in hepatocytes. Besides hepatocytes, HSCs—the main collagen-producing cells in the liver—represent a major driver of fibrosis progression [35]. Thus, we next studied activin A receptor mRNA expression, as well as the effect of activin A and NUCC-555 on HSCs. Since our previous studies [9] demonstrated a strong effect of activin A on cultured rat hepatocytes, we first generated inactive HSCs derived from commercially available activated HSCs and compared their receptor expression with hepatocytes.

**Figure 3:**
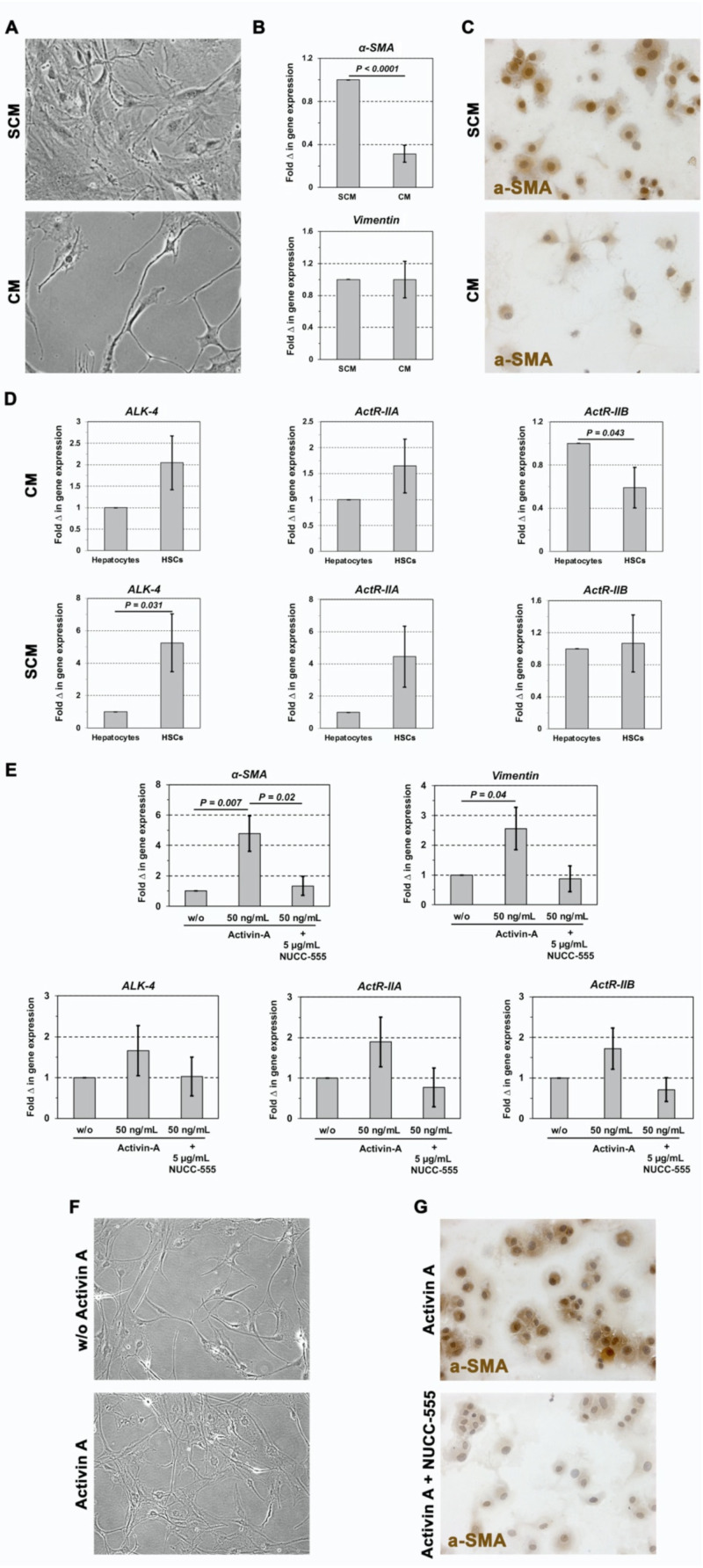
Blocking activin A signaling in HSCs. (**A**–**D**) Characterization of inactivated and activated HCSs. Activated rat HSCs (1 × 10^5^ cells/well in triplicates), cultured in stellate cell medium (SCM) (**A**; upper panel), were inactivated in Clever’s medium (CM) (**A**; lower). (**B**) RNA extracts were analyzed by qRT-PCR and expression levels of selected HSC-specific genes were determined. Mean ± SEM values of 4 independent experiments (including two replicate PCR analyses, each) are expressed as fold changes in inactivated HCSs (in CM) with respect to activated HSCs (in SCM), set at a value of 1. (**C**) Immunocytochemistry for α-SMA on cytospins. (**D**) Activin receptor mRNA expression on inactivated (CM) and activated HSCs (SCM). Mean ± SEM values of 4 independent experiments (two replicate PCR analyses, each) are expressed as fold changes in mRNA expression compared to hepatocytes, set at a value of 1. (**E**–**G**) Blockage of activin A-induced HSC activation by NUCC-555. Activated HSCs were inactivated in CM for 4 days, followed by incubation without (w/o) activin A, with activin A, or activin A and NUCC-555 for 4 days thereafter. (**E**) qRT-PCR analyses for a-SMA, vimentin, and activin receptor mRNA. Mean ± SEM values of 4 independent experiments (two replicate PCR analyses, each) are expressed as fold changes in mRNA expression with respect to inactivated HSCs without any treatment, set at a value of 1. (**F**) Morphology of cultured HSCs in CM without or with 50 ng/mL activin A treatment. (**G**) Immunocytochemistry for α-SMA on cytospins. HSCs were cultured with 50 ng/mL activin A without or with 5 mg/mL NUCC-555. Original magnification, 100× (**F**) and 200× (**A**,**C**,**G**).

Activated rat HSCs cultured in SCM express >90% α-SMA and exhibit a typical fibroblastic morphology in vitro (Figure 3A; upper panel). Based on personal communications with Dr. Michalopoulos, activated HSCs can be deactivated in CM. After culturing activated HSCs in CM for 4 days, we observed a clear change in cell morphology—a characteristic cell shape with long spines was seen (Figure 3A, lower panel). These cells exhibited a 3.2-fold reduced α-SMA expression compared to activated HSCs (Figure 3B, upper panel; Figure 3C), although no changes were detected in the expression levels of *vimentin* (Figure 3B, lower panel), which is expressed in HSCs but not in hepatocytes. qRT-PCR analyses showed higher expression of *ALK-4* and *ActR-IIA* mRNAs in CM-cultured inactivated HSCs compared to hepatocytes but reduced *ActR-IIB* expression (Figure 3D, upper panels). However, mRNA receptor expression was markedly elevated in activated HSCs (SCM) vs. inactive HSCs (Figure 3D, lower panels).

In subsequent studies, we performed qRT-PCR and immunocytochemistry to determine the effect of activin A and its antagonist on inactivated HSCs in vitro. Kiagiadaki et al. [23] cultured HSCs with 100 ng/mL activin A for 24 h; however, no response to activin A was observed. In our studies, we incubated HSCs for 48 h as we did with hepatocytes (see Figure 2), and additionally for 72 h; however, no effect was observed (data not shown). Therefore, we incubated HSCs for 4 days in CM to generate inactive HSCs, followed by administration of 50 ng/mL activin A for 4 days. There was a 4.8-fold and a 2.6-fold increase in *a-SMA* and *vimentin* mRNA, respectively (Figure 3E, upper panels). Inactivated HSCs (Figure 3F, upper panel) switched to a profibrogenic phenotype (Figure 3F, lower panel) with high α-SMA protein expression (3G, upper panel), as we observed in activated HSCs (Figure 3A,C, upper panels). Using qRT-PCR for *ALK-4*, *ActR-IIA*, and *ActR-IIB* mRNA expression, activin A stimulated activin A receptor expression (Figure 3E, lower panels). These activin A-mediated effects were completely blocked in HSC cultures with 5 μg/mL NUCC-555 treatment (Figure 3E). Moreover, immunocytochemical analyses showed a low α-SMA protein expression after activin A antagonist administration (Figure 3G, lower panel), as we observed in inactivated HSCs (Figure 3C, lower panel).

Taken together, these studies demonstrate that activin receptor expression in HSCs is comparable to hepatocytes and sufficient to mediate activation of HSCs after long-term activin A treatment. Importantly, antagonizing activin A signaling, using the novel small-molecule NUCC-555, successfully impairs HCS stimulation.

### 3.4. Accelerated Rat Liver Regeneration through NUCC-555 Administration

**Figure 4:**
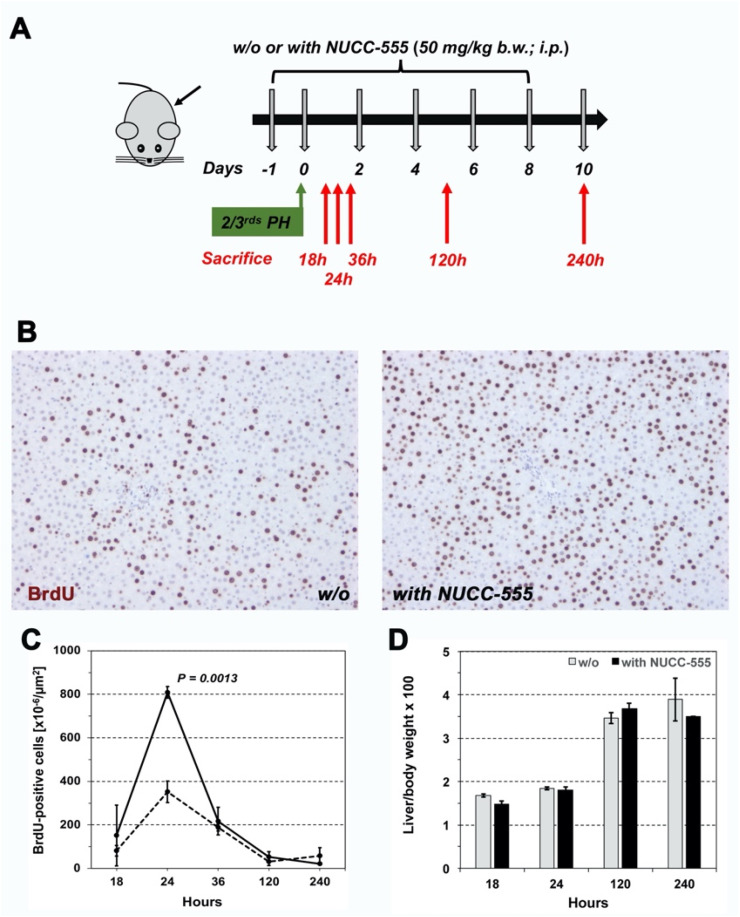
Effect of activin A blockage on rat liver regeneration after PH. (**A**) F344 rats were administered with NUCC-555 (50 mg/kg b.w.) one day prior and immediately after 2/3 PH, followed by treatment on days 2, 4, 6, and 8. Rats were sacrificed at various time points. (**B**,**C**) Nuclear labeling index was determined by standard BrdU staining. (**B**) shows representative images of BrdU-stained liver sections derived from non-treated animals and rats with NUCC-555 administration at 24 h after PH. Original magnification, 100×. (**C**) Effect of NUCC-555 treatment on the time course of hepatocyte proliferation after PH (*solid line*), compared to rats without activin A antagonist (*dotted line*). Values are mean ± SEM of BrdU-positive cells derived from three rats at each time point (10 microscopic fields from 2–3 liver sections per animal were analyzed). (**D**) Liver/body weight ratios in both groups (mean ± SEM).

Activin A is an autocrine growth regulator in the regenerating liver [36]. To study the effect of NUCC-555 in antagonizing activin A signaling in vivo, we performed 2/3 PH in two groups of 7-week-old F344 rats, weighing 151.3 ± 2.1 g. One group received 50 mg/kg b.w. NUCC-555 at one day prior and immediately after PH, followed by treatment on days 2, 4, 6, and 8. Rats were sacrificed at various time points (n = 3 rats/time point) (Figure 4A) and the nuclear labeling index was determined using IHC for BrdU incorporation (Figure 4B shows representative images of BrdU^+^ cells in rats without and with NUCC-555 at 24 h after PH). Increasing changes in nuclear labeling were detected at 18 and 24 h in both groups, which decreased thereafter (Figure 4C). However, a 1.9-fold and a 2.3-fold increase of BrdU^+^ cells was observed at 18 and 24 h in rats treated with NUCC-555 relative to control rats, respectively. No differences were detected at later time points between these groups (Figure 4C). Although significant changes in the proliferation rates between both groups at early time points after PH were observed, no differences in their liver/body weight ratios were detected throughout the observation time (Figure 4D).

### 3.5. Effect of NUCC-555 in Rodent Fibrosis Models

**Figure 5:**
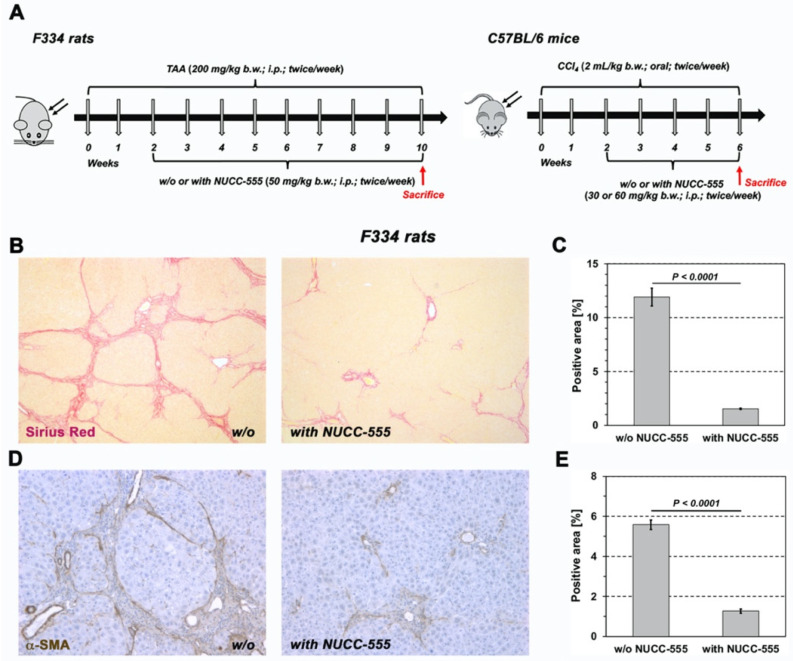

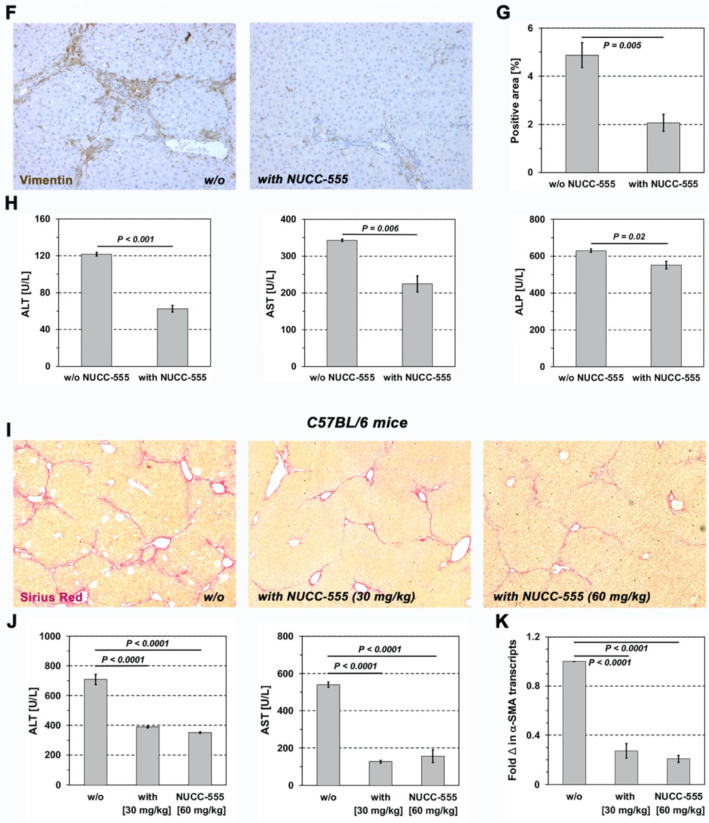
Reduced fibrosis progression in rodent liver with blocked activin A signaling. (**A**) To induce fibrosis in rats (left panel), TAA was injected into mutant DPPIV^–^ F344 rats. Two weeks after starting TAA administration, one group was treated with NUCC-555. TAA administration was continued in rats with and without activin A antagonist treatment, thereafter. Rats were sacrificed at 10 weeks after starting TAA treatment. To induce fibrosis in mice (*right*), C57BL/6 mice were treated with CCl_4_ for 6 weeks. After 2 weeks, NUCC-555 treatment was started in one group. Mice were sacrificed at 6 weeks. (**B**–**H**) ***Rat fibrosis model***. (**B**) Selected areas of Sirius Red-stained tissue sections of rat livers without (*left*) or with (*right*) antagonist treatment. (**C**) Quantification of Sirius Red-stained collagen. (**D**) Immunohistochemical detection of α-SMA-positive cells in rat livers without (*left*) or with (*right*) NUCC-555 treatment. (**E**) Quantification of liver areas with activated HSCs. (**F**,**G**) IHC for vimentin and quantification. (**C**,**E**,**G**) Values are mean ± SEM of three rats without and 4 rats with antagonist administration (10 microscopic fields from 2–3 liver sections per animal were analyzed). Original magnification, 50× (**B**) and 100× (**D**,**F**). (**H**) Blood serum analyses. (**I**–**K**) ***Mouse fibrosis model***. (**I**) shows photomicrographs of Sirius Red-stained liver tissue sections. Original magnification, 50×. (**J,K**) show serum ALT/AST levels and *α-SMA* mRNA expression in mice treated with CCl_4_ with or without NUCC-555 (n = 3–5 mice per group). Data are presented as mean ± SEM.

**Figure 6:**
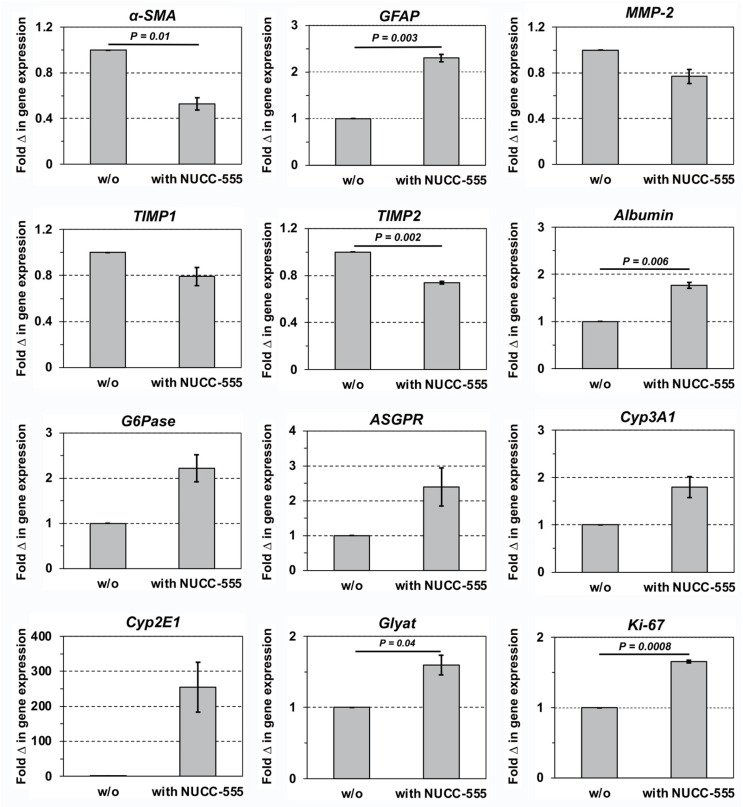
Effect of activin A blockage on mRNA expression during rat fibrosis progression. TAA was injected into mutant DPPIV^–^ F344 rats for 10 weeks. Starting after 2 weeks, one group received NUCC-555 twice a week (see Figure 5A). Liver-derived RNA extracts were analyzed by qRT-PCR analysis for mRNA of genes for stellate cell activation and fibrogenesis, metabolic hepatocyte function, and proliferation. Values are mean ± SEM of liver samples from rats with NUCC-555 administration (n = 4 rats) compared to non-treated rats, set at a value of 1 (n = 3 rats). Two replicative PCR analyses were performed for each gene.

Having demonstrated that NUCC-555 can efficiently block activin A signaling in rat hepatocytes in vitro and in vivo, we next determined whether antagonizing activin A—which is involved in the pathogenesis of several human liver diseases and cancer patients [7,17,18,19,20,21,22,23]—can affect fibrogenesis and the extend of liver fibrosis. To induce fibrosis, 200 mg/kg TAA was injected twice weekly into 2-month-old F344 rats, weighing 237.18 ± 3.68 g. After two weeks, one group received NUCC-555 (50 mg/kg b.w.) twice a week. TAA administration was continued in rats with (n = 3 rats) and without activin A antagonist treatment (n = 4 rats) thereafter (Figure 5A, left panel). At 10 weeks after starting TAA treatment, histological examination of Sirius Red-stained liver sections from rats without NUCC-555 administration showed progressive liver injury leading to moderate fibrosis and beginning cirrhosis, reflected by tissue areas with nodularity caused by fibrous septa bridging portal areas (Figure 5B, left panel). In contrast, only mild fibrosis was observed in activin A-antagonized livers (Figure 5B, right panel). Quantification of Sirius-Red-stained collagen showed 7.9-fold less collagen accumulation in livers of NUCC-555-treated animals compared to control rats (1.5 ± 0.1% vs. 11.9 ± 0.8%; Figure 5C). Moreover, using immunohistochemical analyses for α-SMA and vimentin, a significantly reduced number of activated stellate cells were detected in NUCC-555-treated livers (Figure 5D–G). Moreover, we determined significantly reduced serum ALT, AST, and ALP levels in rats with NUCC-555 administration (Figure 5H). We also performed qRT-PCR analyses to determine the expression levels of genes relevant to fibrosis. We observed a marked decrease in *a-SMA*, matrix metalloproteinase-2 *(MMP-2)*, tissue inhibitor of metalloproteinase-1 *(TIMP1)*, and *TIMP2* mRNA and up-regulation of glial fibrillary acidic protein *(GFAP)* in liver samples of TAA-treated rats with NUCC-555 compared to control rats (Figure 6), indicating a significant reduction of the fibrinogenic process by the activin A antagonist. In addition, liver samples derived from NUCC-555 animals showed higher *albumin*, glucose-6-phosphatase *(G6Pase)*, asialoglycoprotein receptor *(ASGPR)*, cytochrome 3A1 *(Cyp3A1)*, *Cyp2E1*, and glycine-N-acyltransferase *(Glyat)* mRNAs, which are related to hepatocyte-specific cell functions (Figure 6). Moreover, we detected a 1.7-fold increased expression of *Ki-67* mRNA (Figure 6), indicating increased numbers of proliferating cells in NUCC-555-treated rats.

To further study the effect of the activin A antagonist in a murine fibrosis model, 8-week-old C57BL/6 mice were treated with CCl_4_ for 6 weeks. After 2 weeks, NUCC-555 treatment was started (30 or 60 mg/kg b.w.), followed by CCl_4_ administration twice a week (n = 3–5 mice per group; Figure 5A, right panel). NUCC-555-treated animals showed less collagen accumulation (Figure 5I), as well as significantly lower ALT and AST serum levels (Figure 5J) and *a-SMA* mRNA expression (Figure 5K). Taken together, our findings with the small-molecule NUCC-555 represent an efficient strategy to block activin A-mediated events causing liver fibrosis.

## 4. Discussion

Progressive liver injury activates HSCs producing collagen that drives fibrosis, which results in impaired hepatocyte regeneration and function [35]. Fibrosis is a common consequence of many CLDs, the reversal of which remains a major clinical need. In recent studies, we showed that activin A plays a critical role in maintaining normal liver function [9]. There is growing evidence implicating that activin A is involved in the progression of CLDs [7,17,18,19,20,21,22,23]. However, therapeutic targeting of activin A signaling has not been successfully undertaken. In the present study, we evaluated the novel highly specific activin A antagonist NUCC-555 and made three major observations: *First*, we demonstrated that NUCC-555 sufficiently blocks the activin A/p15^INK4b^ axis in hepatocytes. Moreover, we showed that HSCs express all activin A-specific receptors required for activin A-mediated HSC activation. *Secondly*, in partially hepatectomized rats, activin A blockage significantly increases the proliferative activity of hepatocytes. *Third*, small-molecule-based blockage of activin A signaling significantly attenuates fibrosis progression in chemical-induced rodent fibrosis models. These studies clearly demonstrate the beneficial effects of antagonizing activin A, using the novel small-molecule NUCC-555. Our findings represent a novel promising therapeutic strategy to block activin A-mediated events causing liver fibrosis.

Therapeutic targeting of activin A by small-molecules to improve the regenerative capacity and compensate for the loss of functional liver tissue mass represents a new approach with great clinical potential. Zhu et al. identified the small-molecules NUCC-474 and NUCC-555, which specifically bind activin A compared to other TGF-β-superfamily members and sufficiently antagonize activin A in ex vivo ovary cultures and in ovariectomized mice [24]. Because our previous studies demonstrated that hepatocytes are the major activin A-positive cell population in normal liver [9], we tested the cell toxicity of both antagonists in primary rat hepatocytes and human Huh-7 cells, which we used as a reference cell line, previously. Based on the results of our in vitro studies (see Figure 1 and Supplemental Figure S1)—NUCC-474 exhibited higher cytotoxicity—we further investigated the antagonistic effect of NUCC-555 in hepatocytes, as well as its translational potential.

Activin A acts as a prominent regulator of hepatocyte growth inhibition and senescence by upregulating many specific molecules involved in numerous biological processes [9]. Moreover, activin A was identified among multiple senescence-associated secretory phenotype (SASP) factors that mediate paracrine senescence [37]. Our data show (see Figure 2) that up-regulation of the G1 cell cycle inhibitor p15^INK4b^—also a crucial mediator of activin A-induced hepatocyte senescence [9,16]—was 3.8-fold reduced after NUCC-555 treatment. In addition, activin A-induced increase in senescence-related genes, *DEC1* and *Glb1* [38,39] were completely reversed by NUCC-555. Expression levels of other activin A target genes, including *Adam12*, *Pmepa1*, and *Lmcd1*—known to be involved in receptor endocytosis, fibrogenesis, and tumor progression [40,41,42,43]—were significantly elevated by activin A (confirming our previous studies) [9], and entirely antagonized by NUCC-555. Together, these data show that NUCC-555 treatment efficiently blocked paracrine activin A effects in hepatocytes.

After demonstrating the beneficial effects of antagonizing activin A signaling in cultured hepatocytes, we studied the effect of the small-molecule in vivo. Kogure et al. [44] demonstrated the essential role of activin A during the initiation of DNA synthesis after PH. Our studies show that NUCC-555 treatment accelerated hepatocyte proliferation by 100% at 18 and 24 h following PH (see Figure 4C). These results are in accordance with previous studies, wherein a single dose of follistatin given immediately after PH increased hepatocyte proliferation [44]. Interestingly, the observed increase in hepatocyte proliferation was higher in our studies compared to the report by Kogure et al. [44]. Therefore, our data show that antagonizing activin A signaling, using the novel small-molecule NUCC-555, promotes liver regeneration.

Using a CCl_4_-induced rat model of hepatic failure, Nishikawa et al. [45] demonstrated that re-expression of the transcription factor hepatocyte nuclear factor (HNF)-4α restored functional rat liver mass, reversing terminal chronic hepatic failure. Our previous studies identified a top-ranked network of gene connectivity consisting of up-/downregulated focus molecules, which exhibit a direct relationship to the transcription factor HNF-4α to activin A signaling [9]. In addition, we showed that transplanted rat fetal liver stem progenitor cells (FLSPCs) differentiated into hepatocytes and restored an injured liver parenchyma with TAA-induced advanced fibrosis/cirrhosis and exhibited antifibrotic effects [46]. In the present study, we blocked activin A signaling by NUCC-555 administration promoting forced liver regeneration in two rodent models of CLD. Rat and mouse livers without NUCC-555 administration showed progressing fibrogenesis compared to animals with antagonist treatment (see Figure 5B–G,I). The fibrotic liver in non-treated animals exhibited increased *a-SMA*, *MMP-2*, *TIMP1*, and *TIMP2* mRNAs (see Figure 5K and Figure 6), indicating increased numbers of activated HSCs and ongoing fibrogenesis [47], and decreased *GFAP* (see Figure 6), which is down-regulated in activated HSCs in advanced fibrosis [48]. Fibrosis progression was further supported by reduced levels of unique hepatocyte-specific mRNA transcripts (e.g., *albumin*, *G6Pase*, *ASGPR*, *Cyp3A1*, and *Cyp2E1* mRNA) (see Figure 6). Finally, reduced liver function was also reflected by significantly higher ALT, AST, and ALP levels in animals without NUCC-555 administration (see Figure 5H,J). These data demonstrate proof of the principle that blocking activin A signaling by a small-molecule-based antagonist effectively impairs fibrosis progression and improves liver function in two rodent models of CLD. However, since activin A is a multifunctional cytokine, further studies are required to study the possible systemic effects of the activin A antagonist.

**Figure 7:**
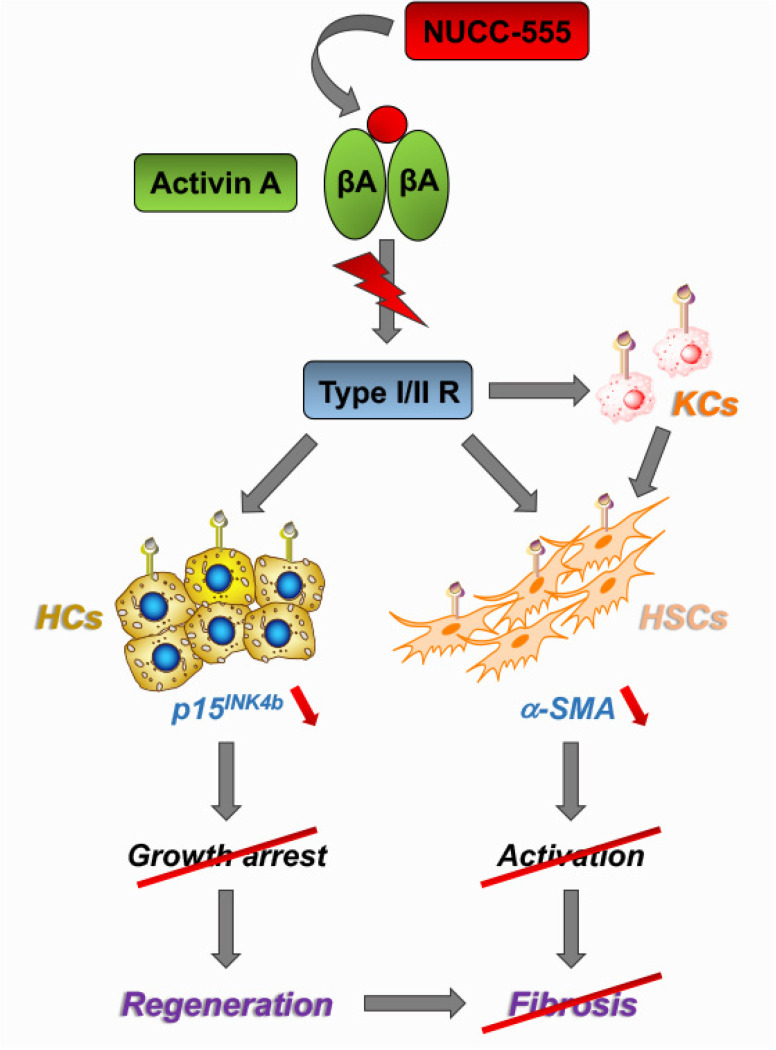
Mechanisms of NUCC-555-mediated effects in blocking liver fibrosis progression. The present studies showed clear evidence that antagonizing activin A signaling in hepatocytes and HSCs promotes liver regeneration and impairs fibrosis progression. Another pathway, in which activin A directly activates Kupffer cells to secrete of TNF-α and TGF-β1 causing HSC activation [23], could also be blocked by NUCC-555. HCs: hepatocytes, HSCs: hepatic stellate cells, KCs: Kupffer cells.

The present studies show that activin A-mediated molecular and cellular functions in hepatocytes can be regulated by NUCC-555, revealing great therapeutical potential. Since HSCs are primary contributors to hepatic fibrosis, we also studied the role of activin A signaling in HSCs. Kiagiadaki and co-workers showed that mouse HSCs do not directly respond to activin A due to the low expression levels of activin A type-II receptors [23]. In contrast, our studies demonstrated that rat HSCs can be activated by activin A treatment in vitro, indicating that the initial level of receptor expression in inactive HSCs is efficient in regulating activin A signaling. Moreover, the authors did not perform additional analyses to measure receptor function (immunocytochemical demonstration of phosphorylation or block experiments with receptor antagonists). Because Kiagiadaki’s and our studies demonstrated similar activin A receptor expression compared to hepatocytes (see Figure 3D), the discrepancies in the obtained outcomes could be the result of different activin A incubation times. Kiagiadaki et al. [23] cultured mouse HSCs with 100 ng/mL activin A for 24 h. In our initial studies, we incubated rat HSCs for 48 h as we did with hepatocytes, and additionally for 72 h, in which we did not observe any response to 50 ng/mL and 100 ng/mL activin A (data not shown). In contrast, after 4 days of treatment, activation of HSCs by activin A was observed, indicating that long-term treatment with activin A is required (see Figure 3E–G). Importantly, our studies clearly demonstrated that activin A signaling in HSCs can be completely inhibited by antagonizing with NUCC-555 (see Figure 3E,G). Besides the observed effect in hepatocytes and HSCs, it cannot be ruled out that activin A signaling was also inhibited by NUCC-555 in other hepatic cell populations. It was reported that activin A induces hepatic fibrosis by stimulating Kupffer cells (KCs) to secrete tumor necrosis factor (TNF)-α and TGF-β1, causing HSC activation [23]. Further in-depth studies are needed to determine the effect of NUCC-555 on KC activation in the fibrotic liver (Figure 7).

Although several antagonists were identified to block activin A signaling, there are currently only a few clinical studies using activin A antagonists in patients. However, these antagonists caused unwanted side effects due to the lack of specificity [49,50]. Small-molecule-based therapeutic strategies have great translational potential in treating CLDs [51]. Our studies show that activin A-induced growth arrest via the activin A/p15^INK4b^ axis can be antagonized by the highly specific small-molecule NUCC-555, promoting liver regeneration. We also demonstrated that the expression of activin A-specific type I and II receptors in HSCs is sufficient to initiate their activation by activin A. Remarkably, activin A-mediated effects in both hepatic cell types were significantly reversed by NUCC-555, causing impairment of fibrosis progression. Therefore, the use of small-molecule-based highly specific activin A antagonists represents a new therapeutic approach and holds great promise for the treatment of patients with end-stage liver diseases.

## Data Availability

Data is contained within the article or Supplementary Materials.

## Supplementary Materials

The following supporting information can be downloaded at https://www.mdpi.com/article/10.3390/cells13070649/s1, Figure S1: Cell toxicity of the activin A antagonist in human Huh-7 cells and primary rat hepatocytes. Table S1: Primer Sequences used for qRT-PCR.

## Abbreviations

HSC, hepatic stellate cell; TGF, transforming growth factor; CLD, chronic liver disease; PH, partial hepatectomy; DPPIV, dipeptidyl-peptidase IV; F, Fisher; NIH, National Institutes of Health; SCM, stellate cell medium; FBS, fetal bovine serum; O/N, overnight; HGM, hormonally defined growth medium; MTT, 3-(4,5-dimethylthiazol-2-yl)-2,5-diphenyltetrazolium bromide; IHC, immunohistochemistry; α-SMA, alpha smooth muscle actin; CM, Clevers’ medium; qRT-PCR, quantitative reverse transcription-polymerase chain reaction; Gapdh, glyceraldehyde 3-phosphate dehydrogenase; BrdU, bromodeoxyuridine; b.w., body weight; TAA, thioacetamide; CCl_4_, carbon tetrachloride; AST, aspartate aminotransferase; ALT, alanine aminotransferase; ALP, alkaline phosphatase; SEM, standard error mean; DEC1; deleted in esophageal cancer 1; Glb1, galactosidase b 1; Adam12; ADAM metallopeptidase domain 12; Pmepa1, prostate transmembrane protein, androgen induced 1; Wnt7a, Wnt family member 7a; Lmcd1, LIM and cysteine-rich domain 1; Shroom4, shroom family member 4; Trpm4, transient receptor potential cation channel, subfamily M, member 4; MMP-2, matrix metalloproteinase-2; TIMP-1/2, tissue inhibitor of metalloproteinase-1/2; GFAP, glial fibrillary acidic protein; G6Pase, glucose-6-phosphatase; ASGPR, asialoglycoprotein receptor; Cyp, cytochrome; Glyat, glycine-N-acyltransferase; HNF, hepatocyte nuclear factor; KC, Kupffer cell; TNF, tumor necrosis factor.
