# Supplementary material for: Antagonizing Activin A/p15INK4b Signaling as Therapeutic Strategy for Liver Disease
Source: Cells. 2024 Apr 8;13(7):649. doi: 10.3390/cells13070649 (PMC11011318; doi:10.3390/cells13070649)
Supplement: Supplementary file 1 [file cells-13-00649-s001.zip › cells-2733815-supplementary.pdf]

SUPPLEMENTAL MATERIAL AND METHODS

Isolation and purification of rat hepatocytes

Rat livers were perfused with 5 mM ethylene glycol-bis(β-aminoethyl ether)-N,N,N',N'-tetraacetic acid (EGTA) solution, followed by 5000 U/100 mL collagenase solution. The perfused livers were then excised, minced, and suspended in Hank's balanced salt solution (HBSS). Hepatocyte suspensions were filtered through an 80-μm nylon mesh and centrifuged for 2 minutes at 50 x g at 4°C. Cell pellets were washed 3 times with Dulbecco's modified eagle medium (DMEM) containing 10% fetal bovine serum (FBS). Cell pellets containing hepatocytes were resuspended and purified through Percoll gradient solution. Aliquots of cell suspensions were mixed with equal volumes of Percoll solution (containing Percoll/10x HBSS, 9:1) and centrifuged for 10 minutes at 50 x g at 4°C. Cells were washed twice with DMEM containing 10% FBS at 50 g for 5 minutes. Cell viability of purified hepatocytes was greater than 90%.

Nuclear staining for BrdU

Formalin-fixed/paraffin-embedded rat liver sections were deparaffinized in xylene and rehydrated, followed by microwave antigen retrieval for 17 min in 10 mM citrate buffer. Afterwards, sections were incubated in avidin/biotin (Vector Laboratories, Newark, CA) and serum-free protein blocking solutions (DAKO, Agilent, Santa Clara, CA), followed by primary antibody solution mouse anti-BrdU (Proteintech, Rosemont, IL). Slides were stained with biotin-conjugated horse anti-mouse IgG, followed by the Vectastain ABC procedure (Vector). Antibody staining was developed using DAB substrate kit (Vector) and counterstained with hematoxylin to visualize the cell nuclei.

Collagen staining

Sirius Red staining of paraffin-embedded rat or mouse liver sections was performed, using a standard technique. Briefly, slides were deparaffinized in xylene and rehydrated. Sections were then incubated utilizing Picro-Sirius Red solution, followed by acidified acid solution (Abcam, Cambridge, UK).

Immunohistochemical/immunocytochemical detection of a-SMA and vimentin

Paraffin-embedded rat liver sections were deparaffinized in xylene and rehydrated, followed by microwave antigen retrieval for 17 min in 10 mM citrate buffer (for vimentin IHC), followed by blocking steps (avidin/biotin and serum-free protein blocking). Mouse anti-α-SMA (Sigma-Aldrich, St. Louis, MO) or rabbit anti-vimentin (Cell Signaling Technology, Danvers, MA) was used as primary antibody. Secondary antibody was a biotin-conjugated horse anti-mouse or horse anti-rabbit IgG (Vector), followed by the Vectastain ABC procedure. Antibody staining was developed using the DAB substrate kit (Vector) and counterstained with hematoxylin.

Cytospins with HSCs were fixed in 4% paraformaldehyde/phosphate-buffered saline (PFA/PBS) solution, followed by incubation in 0.3% triton-X 100/PBS solution for 10 minutes. IHC for α-SMA was continued as described above (blocking steps, etc.).

SUPPLEMENTAL TABLES

Table S1:

Primer Sequences used for qRT-PCR.

Gene	Primer Sequence	Sequence detected
Rat primers		
ActR-IIA	F5'-GGATTGGGAGACTTCCTGC-3'<br>R5'-GGCCACATTCTTCGTGTAAG-3'	104 bp
ActR-IIB	F5'-ACCTCCGTCACCAATGTGG-3'<br>R5'-CAGATCCACTGAGTCTGGAG-3'	87 bp
Adam12	F5'-GCCAGAATATCGACATCAGC-3'<br>R5'-CTGCCTGACTGCAGGATTG-3'	149 bp
Albumin	F5'-GAAGAGTGGGCACCAAGTGT-3'<br>R5'-AGCACACACAGACGGTTCAG-3	97 bp
ALK-4	F5'-GTCAACTGGCAGATACTGAG-3'<br>R5'-CGGTCCTCCAGATGAGAAG-3'	95 bp
ASGPR	F5'-AGTTAGTCGAGTCACAGCTGG-3'<br>R5'-GCAGACGTCATCATTCCAG-3'	500 bp
α-SMA	F5'-TGTGCTGGACTCTGGAGATG-3'<br>R5'-TAGAGGTCCTTCCTGATGTC-3'	429 bp
Cyp2E1	F5'-ACTTCTACCTGCTGAGCAC-3'<br>R5'-TTCAGGTCTCATGAACGGG-3'	874 bp
Cyp3A1	F5'-GGAAATTCGATGTGGAGTGC-3'<br>R5'-AGGTTTGCCTTTCTCTTGCC-3'	329 bp
Dec1	F5'-CAGACTGACAGTGGTCAGAC-3'<br>R5'-TTGTCTTTATTCATCAGAGGCAG-3'	132 bp

G6Pase	F5'-GACCTCCTGTGGACTTTGGA-3'<br>R5'-AGGCTTGGGTTGCTCCTAGT-3'	438 bp
GFAP	F5'-CGCGTCTCAGTTGTGAAGG-3'<br>R5'-ACTGCTCTGAAGGTTAGCAG-3'	428 bp
Glb1	F5'-AGCCATTCCGCTACATCTCG-3'<br>R5'-AGTAACCAAGCGGGTAAGCC-3'	283 bp
Glyat	F5'-CCTTCCCTGCTGGATACAAA-3'<br>R5'-CTTTATGCAGCGTTCGATGA-3'	174 bp
Ki-67	F5'-ATGCGTCTGCAGAGAAGGTT-3'<br>R5'-CTGACTTTGCCCAGAGATGA-3'	121 bp
Lmcd1	F5'-CTCAGGCTGTGACGAGATC-3'<br>R5'-AAATGAAGTGTTTCCGATGCC-3'	80 bp
MMP-2	F5'-AGCTCCCGGAAAAGATTGAT-3'<br>R5'-CCAGAACTTGTCCCCAGAAA-3'	224 bp
p15^{INK4b}/CDKN2B	F5'-TCACCAGACCTGTGCATGAT-3'<br>R5'-AGGCGTCACACACATCCAG-3'	96 bp
Pmepa1	F5'-AGCTGAACCGAGAGTCTG-3'<br>R5'-GCTATAGCAGGTAGCGCTG-3'	131 bp
Shroom4	F5'-GAATGCTCTCAACAGCATCG-3'<br>R5'-CTTCAGCTCTTTGGCATCTG-3'	106 bp
TIMP1	F5'-TCCCCAGAAATCATCGAGAC-3'<br>R5'-ATGGCTGAACAGGGAAACAC-3'	329 bp
TIMP2	F5'-AATTTATCTACACGGCCCCC-3'<br>R5'-TGATGCAGGCAAAGAACTTG-3'	324 bp
Trpm4	F5'-CACAAGCTCGTGACAAGCG-3'<br>R5'-CTCTCTGATCTGTCCCAGC-3'	98 bp
Vimentin	F5'-CTGCAGCTCCTGGATCTCTT-3'<br>R5'-CTTCGCCAACTACATCGACA-3'	400 bp

Wnt7a	F5'-CCCGAACCCTCATGAACTTA-3'<br>R5'-ACGGCCTCGTTGTATTTGTC-3'	181 bp
Gapdh	F5'-GGCATTGCTCTCAATGACAA-3'<br>R5'-ATGTAGGCCATGAGGTCCAC-3'	95 bp

Mouse primers

α-SMA	F5'-CCACCATGTACCCAGGCATT-3'<br>R5'-TGGAAGGTAGACAGCGAAGC-3'	151 bp
18sRNA	F5'-CGCGGTTCTATTTTGTTGGT-3'<br>R5'-AGTCGGCATCGTTTATGGTC -3'	219 bp

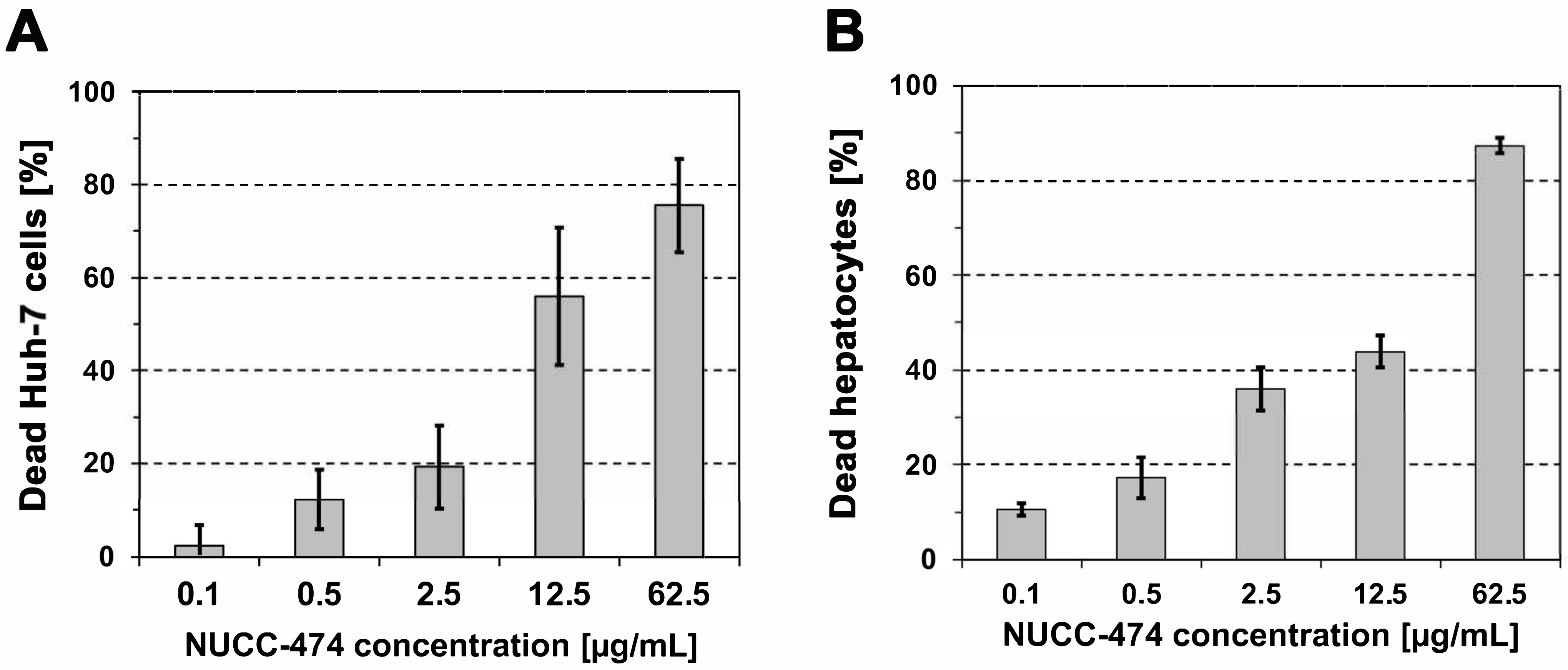

Figure S1. Cell toxicity of the activin A antagonist in human Huh-7 cells and primary rat hepatocytes. Cells were plated on culture plates (1x105 cells/well), followed by incubation with NUCC-474 for 48 hours. Toxicity was determined by standard MTT assay. Cytotoxic effect of NUCC-474 at various concentrations in Huh-7 cells (**A**) and hepatocytes (**B**). Each time point represents the mean ± SEM of three independent experiments, which were performed in triplicates. The percentage of cytotoxicity is normalized to cultured cells without activin A antagonist treatment.
